# EEG synchronization signatures for decoding attentional states during continuous force control

**DOI:** 10.3389/fnins.2025.1654827

**Published:** 2025-10-08

**Authors:** Yilei Zheng, Qiaoxiu Wang, Qianqian Tong, Bohao Tian, Peng Su, Yonghong Xu, Dangxiao Wang

**Affiliations:** ^1^College of Mechanical and Electrical Engineering, Beijing Information Science and Technology University, Beijing, China; ^2^Qiyuan Laboratory, Beijing, China; ^3^Peng Cheng Laboratory, Shenzhen, China; ^4^State Key Laboratory of Virtual Reality Technology and Systems, Beihang University, Beijing, China

**Keywords:** mind wandering, attentional state, electroencephalogram (EEG), neural synchronization, machine learning, force control

## Abstract

**Introduction:**

Mind wandering, the shift of attention from an ongoing task to task-unrelated thoughts, is a pervasive cognitive phenomenon often accompanied by detrimental consequences for task performance. While extensively studied in visual and auditory paradigms, attentional fluctuations during visuo-haptic tasks, such as force control, remain underexplored despite their high relevance to real-world skilled activities such as surgical operations or robotic-assisted manipulation. There exists a critical deficiency in exploring signatures of mind wandering from the perspective of neural synchronization.

**Methods:**

This study investigated EEG-based synchronization features to decode attentional states during a novel continuous force control task using the thought-probe method. Nine healthy male participants tracked a dynamically varying target force while scalp EEG and high-frequency force data were recorded synchronously. EEG epochs preceding self-reported attentional probes were labeled as on-task or mind-wandering states. Spectral power and three synchronization features – cross-frequency coupling, functional connectivity, and neural-behavioral synchronization – were extracted and compared between on-task and mind-wandering states.

**Results and discussion:**

Results revealed that the mind-wandering state was characterized by increased alpha power (8-10 Hz) over frontal-posterior regions and reduced occurrence of high alpha-theta harmonic ratios. It also exhibited increased functional connectivity within sensorimotor networks and decreased mutual information between frontal EEG activity and force errors. Support vector machine classifiers for the binary attentional-state classification, utilizing combined spectral power and synchronization features, achieved 75.53% within-participant and 71.57% cross-participant accuracy, outperforming single-feature models. These findings highlight EEG synchronization signatures of mind wandering and demonstrate their feasibility for decoding attentional states during the force control task. This work may provide a foundation for future exploration of haptic-based neurofeedback systems, which could potentially complement existing visual and auditory modalities in applications such as neurocognitive rehabilitation or skilled motor training.

## 1 Introduction

Sustained attention, the cognitive ability to maintain focus on a given task over extended periods, is essential for our everyday lives. However, this ability is inherently limited. Attention often shifts from the ongoing task to spontaneous, task-unrelated thoughts—a ubiquitous phenomenon termed mind wandering (MW) in academic research (Smallwood and Schooler, 2015; Christoff et al., 2016). While MW may benefit creativity and problem-solving under certain conditions, its negative outcomes, such as impaired task performance, increased risk of accidents, and affective dysfunction, have attracted significant research attention (Killingsworth and Gilbert, 2010; Schooler et al., 2011). Consequently, developing reliable and objective methods for detecting MW has become a major research focus in recent decades (Fortenbaugh et al., 2018; Kam et al., 2022; Tang and Li, 2024). Investigating the neural mechanisms underlying MW and establishing objective detection methods hold promise for the diagnosis and intervention of attention-related neurological disorders, such as mild cognitive impairment (MCI) and attention deficit hyperactivity disorder (ADHD) (Zhao and Yuan, 2025; Wiebe et al., 2024).

The MW has primarily been studied through vigilance tasks within visual or auditory modalities, such as the continuous performance task (CPT), where participants respond to infrequent target stimuli (Esterman et al., 2013), and the sustained attention to response task (SART) which typically requires responding to frequent non-targets while withholding responses to rare targets (Eskandari Nasab et al., 2024). However, relatively little is known about MW during haptic or visuo-haptic tasks that involve continuous motor regulation, such as force control. Force control refers to the precise and continuous adjustment of muscle output or tool-applied forces to achieve task goals (Nakahara et al., 2002). This ability is critical in many real-world scenarios, such as surgical tool manipulation, robotic-assisted rehabilitation exercises, and sports requiring fine motor adjustments. Studying MW in these contexts is important because lapses in attention can directly impair task performance, leading to increased errors or reduced efficiency.

Although the ultimate goal of MW detection research is to develop objective detection methods for MW, thought probing remains essential due to its inherently subjective nature. A widely used approach involves inserting probe questions into the ongoing task randomly or at the end of each block (Cheyne et al., 2009; Stawarczyk et al., 2011; Seli et al., 2018). Upon encountering a probe, participants are asked to report the content of their thoughts or rate their attentional focus. By comparing measures within the few seconds preceding mind-wandering reports with those preceding on-task reports, researchers have linked behavioral indicators (e.g., errors and response time variability) (Esterman et al., 2013; Zheng et al., 2019; Peng et al., 2021) and physiological signals (e.g., eye movements, pupillometry, and heart rate) (Smallwood et al., 2007, 2011; Wainstein et al., 2017) to self-reported MW.

Regarding neural correlates of attentional fluctuations, the electroencephalogram (EEG) has been widely used due to its high temporal resolution and applicability. EEG power-based metrics and event-related potential (ERP) components have been extensively studied as important parameters for characterizing MW (Kam et al., 2022). Numerous studies have reported reduced amplitudes of ERP components (P1 and P3) prior to performance errors or MW reports (Braboszcz and Delorme, 2010; Kam et al., 2011; Liu et al., 2021), supporting the “perceptual decoupling” hypothesis (Schooler et al., 2011; Smallwood, 2013). This theory posits that attention disengages from external sensory input during MW. Additionally, spectral features including delta, theta, alpha, and beta power have been extensively examined in relation to MW. For example, increased power in the alpha band has been linked to both vigilance decrement and the occurrence of MW during SART (Compton et al., 2019; Jin et al., 2019). Power-based indices derived from spectral bands (e.g., beta-to-alpha ratio and inverse alpha power) have also shown significant correlations with behavioral markers of attentional lapses (Coelli et al., 2018), such as variations in mean reaction time.

While these spectral and ERP features provide valuable markers of MW, they largely capture localized neural activity. To further understand how distributed neural systems coordinate during attentional fluctuations, a growing number of studies have explored neural synchronization-based measures, including cross-frequency coupling (e.g., alpha-theta phase synchrony and harmonicity) and functional connectivity between brain regions. Functional connectivity (FC) measures the synchrony between signals recorded from different electrodes or regions (e.g., via phase-locking value, coherence), reflecting functional integration within networks (Aydore et al., 2013). Numerous studies using functional Magnetic Resonance Imaging (fMRI) have shown that the Default Mode Network (DMN) exhibits increased activation and altered connectivity patterns with other networks (e.g., the dorsal attention network) during the MW state (Jang et al., 2011; Mittner et al., 2014; Christoff et al., 2016; Kucyi et al., 2016). A pioneering study combining DMN activation with pupil diameter achieved promising MW classification accuracy (~80% within-participant, ~65% across-participant) (Groot et al., 2021). Cross-frequency coupling (CFC), which examines synchronization between neural oscillations of different frequencies, is believed to underlie complex information processing and communication. For example, increased alpha-theta phase synchrony has been associated with the occurrence of MW during breath-focused meditation (Rodriguez-Larios and Alaerts, 2019, 2021; Rodriguez-Larios et al., 2020). Despite these advances, the relationship between EEG-derived synchronization features (both FC and CFC) and attentional fluctuations remains less well-established and understood. Few studies have explicitly examined the efficacy of EEG synchronization features in classifying attentional states, and most have focused on visual paradigms (Melinscak et al., 2016; Compton et al., 2019; Groot et al., 2021). The potential of these synchronization features for MW detection is largely unexplored.

Given these limitations, an important next step is to evaluate whether such synchronization features can enhance MW detection when combined with machine learning approaches. Recently, machine learning techniques have increasingly been applied to classify attentional states. The majority of these studies have extracted features from EEG recordings during visual tasks (e.g., SART, visual search tasks) and used spectral power or ERP components as classifier inputs, typically reporting classification accuracies of 60–70% (Jin et al., 2019, 2020; Dong et al., 2021). Beyond basic features, researchers have also explored complexity-based metrics, such as sample entropy and permutation entropy. These metrics, which capture the irregularity or predictability of EEG signals, have yielded promising classification performance (e.g., AUC up to 0.71) in SART paradigms (Chen et al., 2022). A recent study comparing complexity features with traditional band power reported comparable performance (AUC = 0.64) in video learning tasks, with slight improvements from combining features (AUC = 0.66) (Tang and Li, 2024). Notably, features capturing neural synchronization, such as functional connectivity and alpha-theta phase synchrony, represent a promising yet underexplored avenue for improving MW detection accuracy.

Collectively, despite growing interest and recent advances in understanding attentional fluctuations and detecting MW, several critical issues need to be considered, particularly regarding task modalities and feature types. First, the majority of MW research has relied on visual or auditory tasks. In contrast, MW detection in haptic or visuo-haptic tasks—such as force control—remains significantly understudied. The neurocognitive basis of haptic perception involves complex feedback loops from hand tactile receptors to the primary somatosensory cortex (S1) and broader attentional networks (Lederman and Klatzky, 2009; Grunwald, 2008). The hand’s acute perception and force control abilities make it particularly well-suited for investigating attentional fluctuations. Although preliminary work by Peng et al. (2021) using a discrete force control task identified behavioral markers (e.g., reaction time variability) and basic EEG features (e.g., increased frontal-central alpha power) potentially related to MW, a systematic investigation into the neural signatures of attentional states—particularly regarding synchronization dynamics during the force control process —remains lacking. Second, the very few EEG studies examining attentional states during force control tasks have confined analysis to fundamental, well-established metrics such as spectral power density and ERPs (Zhang et al., 2023; Delisle-Rodriguez et al., 2023). There exists a critical deficiency in examining features from the perspective of neural synchronization, including FC, CFC, as well as the synchronization between neural activities and behavioral data, within the haptic modality. These synchronization metrics may reveal how distributed brain networks coordinate and how brain activity interacts with motor output during attentional lapses in force control. They may serve as novel and more sensitive biomarkers compared to isolated power or ERP components. Finally, as a consequence of these gaps, the utility and effectiveness of EEG synchronization features for decoding attentional states during force control tasks remain largely unexplored. Whether robust synchronization signatures can be extracted from complex sensorimotor tasks, and whether these features can reliably distinguish MW from on-task states, remain unclear.

Therefore, this study aims to address these issues by investigating EEG synchronization signatures within a force control paradigm. We propose a novel continuous visuo-haptic force control task in which participants precisely modulate their force to track a dynamically changing target force based on visual cues. During the task, participants reported their attentional states when thought probes appeared. Simultaneously, EEG and high-frequency force data were recorded. The objectives of this study are twofold:

(1) To examine EEG synchronization metrics—specifically cross-frequency coupling, functional connectivity, and neural-behavioral synchronization—sensitive to attentional fluctuations (on-task versus MW states) during force control.
(2) To evaluate the feasibility and classification performance of these synchronization features, both individually and in combination with other metrics, for classifying on-task and MW states using machine learning.

We hypothesize that synchronization metrics, capturing the interactive dynamics within the brain and between the brain and behavior, will provide unique and complementary information for decoding attentional states during force control. By identifying EEG synchronization markers of MW in this underexplored haptic context and evaluating their feasibility for decoding attentional states, this study aims to deepen our understanding of the neural basis of attention and lay the groundwork for future research into developing haptic-based neurofeedback attention training systems. Such systems could potentially offer a valuable complement to existing visual and auditory approaches for neurocognitive rehabilitation or skilled activities training.

## 2 Methods

### 2.1 Participants

Fourteen healthy male adults (mean age = 27.4 ± 3.5 years) participated in the experiment. Informed written consent was obtained from all participants. One participant was excluded for not completing the entire experiment, resulting in a final sample of 13 participants. All participants reported normal or corrected-to-normal vision and were right-handed. The study was approved by the Biological and Medical Ethics Committee of Beihang University and was conducted in accordance with the World Medical Association Declaration of Helsinki.

### 2.2 Task and experimental procedure

**Figure 1:**
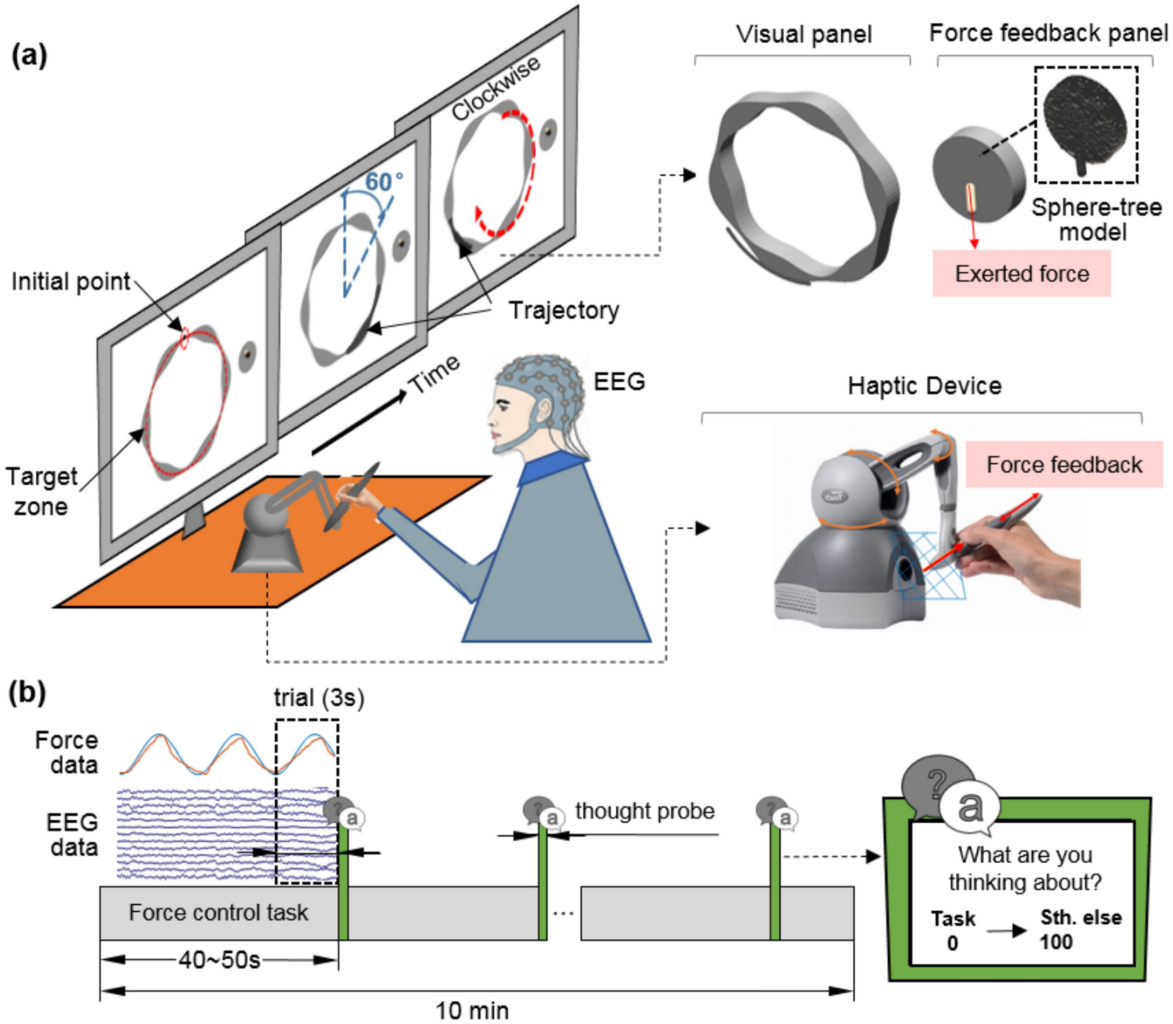
Task design and experimental procedure. **(a)** Continuous force control task. Participants were instructed to adjust the width of a clockwise moving trajectory by modulating their output force, aiming to match the gray target zone as closely as possible. **(b)** Experimental procedure. Force data and EEG data were recorded during the entire experiment. With an interval of 40–50s, a thought probe would appear on the screen, requiring participants to report the degree to which their thoughts wandering from the task. The 3-s data preceding each thought probe was extracted as a single trial.

Considering that humans are more susceptible to attentional lapses during prolonged continuous tasks than discrete ones (Rahman et al., 2021; Reteig et al., 2019), we designed a novel continuous force control task to induce mind wandering, instead of using the discrete paradigms from prior studies (Peng et al., 2021; Zhang et al., 2023). Herein, participants held a pen-shaped handle to exert force while tracking a periodically varying target force. As shown in Figure 1a, participants were seated in front of a computer screen and held the handle with their dominant hand. They maintained a naturalistic pen-holding posture, mimicking handwriting. The handle was an end effector of a haptic device (Touch, 3D Systems Inc., United States). The haptic device allowed six degrees of freedom in movement and three degrees of freedom in force feedback within a 26.5 × 24.1 × 8.9 cm workspace. A virtual 3D scene was constructed, displaying a gray circular object, an annular object, and a pen-shaped object. The circular and annular objects remained fixed, while the pen-shaped virtual object was attached to the real-world handle. As participants moved the handle in the real space, the virtual pen synchronously performed the same movement on the screen.

During the task, a black dot moved clockwise around the gray annular region at a constant speed of 20°/s. The diameter of the black dot (*D*_o_, in millimeters, mm) scaled proportionally to the contact force (*F*_o_, in newtons, N) exerted by the user’s virtual pen on the circular palette, as shown in Equation 1:


(1) $$D_{o}=k\cdot F_{o}+0.5$$

where *k* denotes a scaling constant (*k* = 3 mm/N). This value was empirically determined to ensure that the dot’s size varied within a perceptually noticeable but not overly intrusive range (approximately 2.0–10.0 mm) across the allowable force range of 0.5–3.17 N. A linear mapping was adopted due to its intuitiveness, allowing participants to quickly learn the relationship between applied force and visual feedback. This helped reduce the learning burden and enabled participants to focus on force output regulation. The width of the gray annular region varied nonlinearly over each 60-degree cycle. Participants were required to adjust their exerted force based on the dot’s position to align its trajectory with the target zone. The circular palette was established using sphere tree models with Solidworks (Dassault Systems Inc., United States) and 3D Studio Max (Autodesk Inc., United States). Real-time force feedback was implemented using a validated haptic rendering algorithm (Wang et al., 2013). Exerted forces were recorded at a 1,000 Hz sampling rate using the haptic device and custom scripts developed with Microsoft Foundation Classes (MFC).

The experimental procedure is illustrated in Figure 1b. Each participant completed three sessions of the force control task following a practice session. Each session lasted approximately 10 min, with a short break (1–2 min) to eliminate muscle fatigue. Participants were instructed to focus on the moving dot and modulate their exerted force to match the target zone’s width. At random intervals (40–50 s), a probe question appeared on the screen asking ‘What are you thinking about? Task or Something else?’ Participants rated their attentional states on a 0–100 scale, where 0 indicated being completely focused on the task and 100 indicated complete distraction (Kucyi et al., 2016). EEG and force data from the 3-s time window preceding each thought probe were extracted as individual trials for subsequent analyses.

### 2.3 Behavioral data analysis

**Figure 2:**
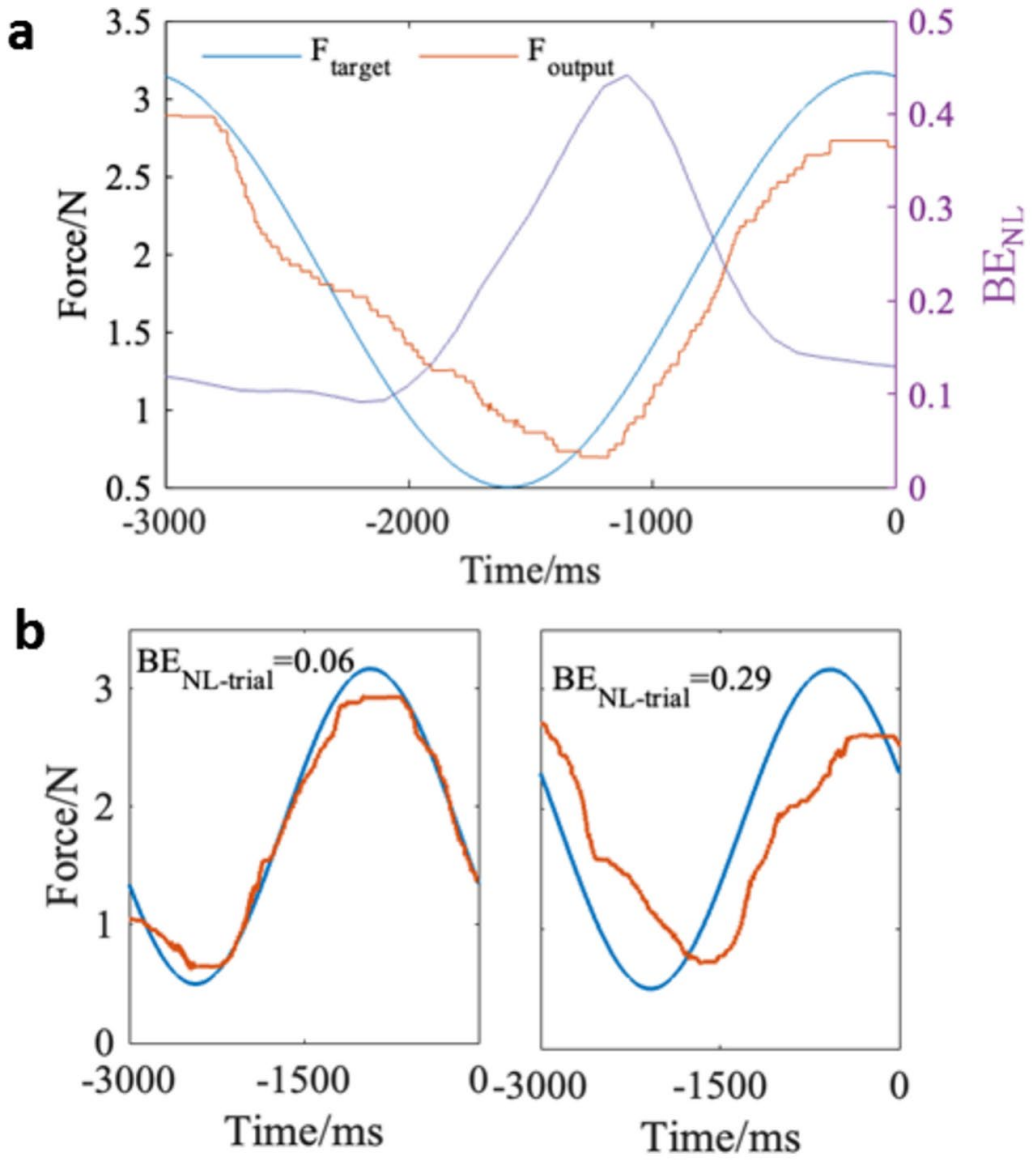
Behavioral performance during the force control task. **(a)** Example data of the target force, output force, and the force error *BE*_NL_ within a trial. **(b)** Examples of the overall force error *BE*_NL-trial_ during two trials. The left panel with a lower value of *BE*_NL-trial_ (0.06) shows relatively good performance and the right panel with a higher value of *BE*_NL-trial_ (0.29) shows relatively poor performance.

During the continuous force control task, the exerted force data were recorded at a sampling rate of 1,000 Hz. Because the target force (*F*_target_), corresponding to the width of the target zone, varied nonlinearly over a 3-s cycle (i.e., 60 degrees), the target force pattern in the 3 s preceding each probe was consistent across trials. As shown in Figure 2a, we calculated the force error within the 3-s period to assess the performance of force control for each trial. The force error (*BE*_NL_) represented the relative difference between the output force (*F*_output_) and the target force (*F*_target_). It was computed at each sampling point and analyzed using sliding windows (1-s length, 90% overlap) to quantify the variation of force errors within each trial. *BE*_NL_ was computed as Equation 2:


(2) $$BE_{\mathrm{NL}}=\sqrt{\frac{\sum {\left(F_{\text{target}}-F_{\text{output}}\right)}^{2}}{\sum {F_{\text{target}}}^{2}}}$$

where the value of *BE*_NL_ ranges from 0 to 1, with smaller values indicating better task performance. We also calculated the sum of *BE*_NL_ within each trial, termed *BE*_NL-trial_, to assess the overall behavioral performance. Figure 2b shows examples of *BE*_NL-trial_ values for two trials, where a smaller value indicates better force control performance.

Additionally, based on the participants’ self-reported ratings, trials were sorted by quartiles for each subject. Specifically, trials were divided into four groups based on the quartiles of the rating scores. Trials with ratings in the lowest quartile (≤25%) were classified as task-focused (onT), and those in the highest quartile (≥75%) as mind-wandering (MW).

### 2.4 EEG data acquisition and analysis

#### 2.4.1 EEG recording and pre-processing

EEG data were recorded from 64 Ag/AgCl electrodes positioned according to the international 10–20 system, using an NSW364 wireless amplifier (Neuracle Technology Co., Ltd., China) at a sampling rate of 1,000 Hz. The impedance of the electrodes was maintained below 5 kΩ using NaCl-based conductive gel (Liu et al., 2019). The reference electrode was placed at the CPz. EEG recordings were synchronized to the force control task, and event times (thought probe onsets) were automatically documented with markers in the continuous EEG data files.

Raw EEG data were preprocessed offline using EEGLAB v13.6.5b (Delorme and Makeig, 2004), an open-source toolbox running in MATLAB R2021a (MathWorks Inc., United States). The EEG data were first bandpass filtered between 2 and 45 Hz using a zero-phase FIR filter with a Hamming window (function *pop_eegfiltnew*). The filtered data were re-referenced using the REST toolbox (Dong et al., 2017). For each participant, data segments within each trial were extracted and concatenated for further analysis. Subsequently, artifact rejection was performed on the concatenated EEG data in three steps. First, bad channels were identified and removed using the *pop_rejchan* function in EEGLAB based on probability, resulting in the removal of an average of 1.0 (SD = 1.15) channels per participant. Second, the remaining data were decomposed using logistic infomax Independent Component Analysis (ICA; function *pop_runica*), and artifact components were identified and rejected using the MARA plugin (Winkler et al., 2011, 2014). Third, the signals were back-projected to the sensor level, and any rejected bad channels were interpolated using the *pop_ interp* function. Finally, the cleaned EEG data for each participant were categorized into two separate sets: on-task (onT) or mind-wandering (MW).

It should be noted that after the ICA-based artifact removal, EEG data from nine participants were included in the subsequent analysis; data from the other four participants were excluded due to extensive artifacts exceeding ±150 μV (Zhang et al., 2019; Peng et al., 2021).

#### 2.4.2 Feature extraction

##### 2.4.2.1 Spectral power

At each electrode, a short-time Fourier transform was performed on the preprocessed EEG data to estimate the power spectrum for each trial. The sliding window length was set to 1 s with 90% overlap between successive segments. Subsequently, EEG power spectra were extracted into five frequency bands: delta (1–4 Hz), theta (4–8 Hz), alpha (8–13 Hz), beta (13–30 Hz) and gamma (30–45 Hz), and log-transformed. For each condition, the spectral powers of all trials were averaged to obtain the mean band power for each frequency band at each electrode. The group-level average power for each frequency band was calculated by averaging the band power across all participants.

##### 2.4.2.2 Cross-frequency coupling: alpha-theta ratios

Given reports of increased alpha-theta harmonicity and phase synchrony during MW, this study assessed alpha-theta cross-frequency coupling during the force control task. Following previous studies (Rodriguez-Larios and Alaerts, 2021), we used the findpeaks approach to compute the cross-frequency ratios between the theta and alpha bands. Specifically, after applying short-time Fourier transformations (sliding window = 100 ms) to the preprocessed EEG data to compute the time-varying spectrum between 4 and 13 Hz, we applied the *findpeaks* function to detect transient peak frequencies in the theta (4–8 Hz) and alpha (8–13 Hz) bands separately. When more than one peak was detected, the frequency with the highest amplitude was selected as the peak frequency. The algorithm detected at least one peak in 99.52% (SD = 0.20%) of alpha-band time points and 94.11% (SD = 1.19%) of theta-band time points. The identified transient peak frequencies in the alpha and theta bands were used to compute their numerical ratio per time point. To analyze the distribution of these ratios, we binned them in steps of 0.1 across a range of 1.1–3.3 and calculated the proportion of time points falling into each bin. Proportions of cross-frequency ratios were computed per epoch and averaged within the same condition (onT or MW) for each participant and electrode. This yielded a normalized distribution across ratio bins for each condition, participant, and electrode, which we refer to as the probability density of alpha-theta ratios. In this framework, a higher probability density at a specific ratio indicates that the alpha-theta system was more likely to align around that harmonic relationship during the task. Neurophysiologically, this suggests that certain stable harmonic states are more prevalent, which may reflect a mechanism that facilitates efficient cross-frequency synchronization necessary for sustained attention (Rodriguez-Larios and Alaerts, 2019).

##### 2.4.2.3 Functional connectivity: within-band coupling between electrode pairs

Phase Locking Value (PLV) quantifies phase synchronization between electrode pairs by measuring the absolute value of the mean phase difference between two signals as a complex unit-length vector (Rosenberg et al., 1989; Aydore et al., 2013). PLV is a measure of pairwise functional connectivity commonly used to quantify the phase coupling between two nonlinear signals. It has a range from 0 to 1, where a value of 0 indicates no phase coupling and a value of 1 indicates complete phase locking. In this study, we computed sensor-level PLV between all electrode pairs using the *ft_connectivityanalysis* function from the FieldTrip toolbox (Oostenveld et al., 2011), generating five 64 × 64 connectivity matrices (one matric per band). To assess their significance, we employed the Network-Based Statistic toolbox to perform permutation testing on the connectivity matrices (Zalesky et al., 2010). This approach allows us to control for family-wise error rates while identifying significant network components. We also extracted community connectivity within and across distinct brain regions and performed repeated one-way analysis of variance (ANOVA) to assess the significance of connectivity patterns among predefined brain communities.

##### 2.4.2.4 Neural-behavioral synchronization: mutual information

Mutual information (MI) between EEG power amplitude and force error was computed to quantify the neural-behavioral synchronization. MI (*X*, *Y*) indicates both linear and nonlinear statistical dependencies between two variables *X* and *Y*, which can be computed as Equation 3:


(3) MI(X,Y)=H(X)+H(Y)−H(X,Y)

where *H*(X) and *H*(*Y*) denote the priori uncertainty of *X* and *Y*, respectively. *H*(*X*, *Y*) denotes the posteriori uncertainty on *X* when the measurement of *Y* is given. The value of MI(*X*, *Y*) answers the question: “Given a measure of *Y*, how many bits of information about *X* can be predicted on average?” (John et al., 2018; Liang et al., 2022). In this study, we computed MI between the within-trial force error (*BE*_NL_) and the corresponding EEG power amplitude (obtained using the short-time Fourier transform) for each electrode. For each subject, the MI values formed a multidimensional array with dimensions: frequency point × electrode × trial.

### 2.5 Features selection

All the behavioral and EEG features were first compared between conditions. To identify significant effects, we employed appropriate statistical tests (*t*-tests or cluster-based permutation testing), and only these significant features were retained for subsequent classification. Specifically, for the behavioral force errors, data were first averaged across trials for each condition within subjects, and then paired-sample *t*-tests were performed to evaluate condition-related differences. For EEG features (i.e., spectral power, alpha-theta ratios, PLV, and MI), cluster-based permutation testing was adopted to evaluate condition-related differences. This nonparametric statistical approach controls the family-wise type I error rate that arises from multiple comparisons across electrodes and frequency bins by employing Monte Carlo randomization. Briefly, the data were shuffled (1,000 permutations) to estimate a null distribution of effect sizes based on cluster-level statistics—specifically, the sum of *t*-values with the same sign across adjacent electrodes, frequencies, or ratios. The cluster-corrected *p*-value was defined as the proportion of permuted datasets in which the cluster-level statistic exceeded that of the original data (cluster-defining threshold: *p* < 0.05). Statistically significant features and electrodes were then selected as the final feature subset and used as inputs for the classifier. We chose cluster-based permutation testing because this nonparametric method can effectively control false positives under multiple comparison corrections while accounting for the spatial and spectral contiguity of EEG data (Maris and Oostenveld, 2007). This approach yields interpretable clusters of features rather than isolated points, which is particularly appropriate for EEG and connectivity analyses (Pernet et al., 2015).

### 2.6 Classifier training and validation

Using the identified features as inputs and the attentional state labels (onT or MW) as outputs, we trained the classifiers using the support vector machine (SVM) algorithm to decode attentional states for each trial. Although previous studies have employed various machine learning algorithms, such as decision trees (Tasika et al., 2020), random forests (Chen et al., 2022), and artificial neural networks (Hosseini and Guo, 2019), this study applied SVM due to its suitability for small-sized datasets with low-dimensional data. Moreover, most prior studies on attention decoding have reported superior classification performance using SVM (Jin et al., 2019, 2020; Dong et al., 2021; Jothiral et al., 2025). A radial basis function (RBF) was selected as the kernel function, and the default parameter settings in LIBSVM were applied (i.e., penalty parameter C = 1 and kernel parameter *γ* = 1/feature dimension). To ensure comparability across features, all input features were z-score standardized within each training fold before model fitting, and the same transformation was applied to the corresponding test fold.

Two cross-validation strategies were employed to evaluate the trained models:

(1) Leave-one-subject-out (LOSO) cross-validation for cross-participant evaluation: in each iteration, one participant’s data were used as the test dataset, while data from the remaining *N*-1 participants formed the training dataset. This process was repeated *N* times, where *N* denotes the number of participants included in the analysis (*N =* 9). The mean classification performance was then averaged across all iterations.
(2) Five-fold cross-validation for within-participant evaluation: for each participant, trials were randomly divided into five subsets while preserving class balance (i.e., each fold contained equal numbers of onT and MW trials). In each round, one subset served as the test dataset, while the remaining four subsets were used for training. The average classification performance was computed across all folds and all participants.

Finally, we computed five commonly used metrics—accuracy, recall, precision, F1-score, and the area under the receiver operating characteristic curve (AUC)—to comprehensively evaluate the effectiveness of the selected features in classifying attentional states. The classification performance for various feature combinations was also assessed using these metrics.

## 3 Results

### 3.1 Self-reported ratings and behavioral performance

**Figure 3:**
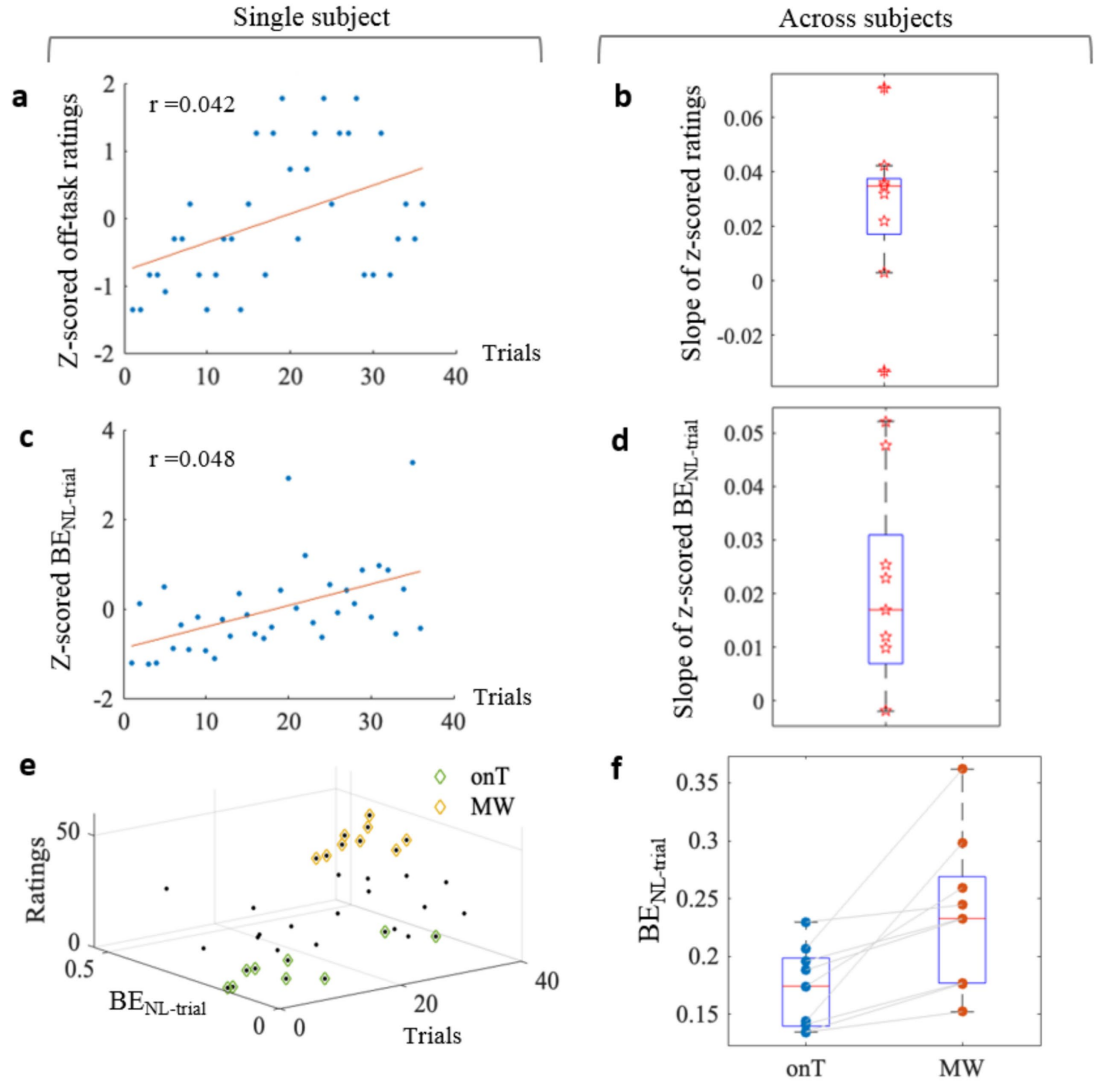
Behavioral measures of attentional states. **(a)** *Z*-scored off-task ratings (blue dots) and linear regression fit (red line) for a single representative participant. **(b)** Group-level slope coefficients derived from the linear regression of z-scored off-task ratings. **(c)** *Z*-scored trial-level force error (*BE*_NL-trial_; blue dots) and linear regression fit (red line) for the representative participant. **(d)** Group-level slope coefficients for z-scored *BE*_NL-trial_. **(e)** Trial classification based on rating quartiles (onT: green; MW: yellow). **(f)** Comparison of force error between onT and MW conditions.

To assess whether the off-task ratings reported by participants increased over time during the force control task, we performed a linear regression analysis on the z-scored ratings across trials for each participant. Figure 3a illustrates the rating scores of a representative participant across all trials (blue dots), along with the corresponding regression-fitted curve (red line). Figure 3b presents the group-averaged slope values of the regression-fitted curves. A one-tailed one-sample *t*-test confirmed that the slope was significantly greater than zero [*t*(8) = 2.53, *p* = 0.018], indicating a significant upward trend in self-reported off-task ratings over time. For the trial-level force error (i.e., *BE*_NL-trial_), we performed a similar linear regression on the z-scored values across trials for each participant. Figure 3c shows the *BE*_NL-trial_ values of a representative participant with the corresponding regression-fitted curve, and Figure 3d summarizes the slope values for all participants. A one-tailed one-sample *t*-test on these slope values revealed a significant increasing trend in force error over trials (*t*(8) = 3.16, *p* = 0.007). The *z*-scored MW ratings and *BE*_NL-trial_ data for all participants are provided in Supplementary Figures 1, 2, respectively.

During the experiment, seven participants completed 36 trials and the other two participants completed 48 trials. As shown in Figure 3e, trials were classified based on rating quartiles: the trials in the lowest quartile (≤25%) were labeled as onT condition, and those in the highest quartile (≥75%) as MW condition. Consequently, two participants contributed 12 trials per condition, while the remaining seven participants contributed 9 trials per condition. Group-averaged force errors (*BE*_NL-trial_) for the two conditions are presented in Figure 3f. Paired-sample *t*-tests revealed significantly higher force errors during the MW condition (0.24 ± 0.07) compared to the onT condition [0.17 ± 0.03; *t*(8) = 3.44, *p* = 0.004].

### 3.2 Condition-specific differences in spectral power

**Figure 4:**
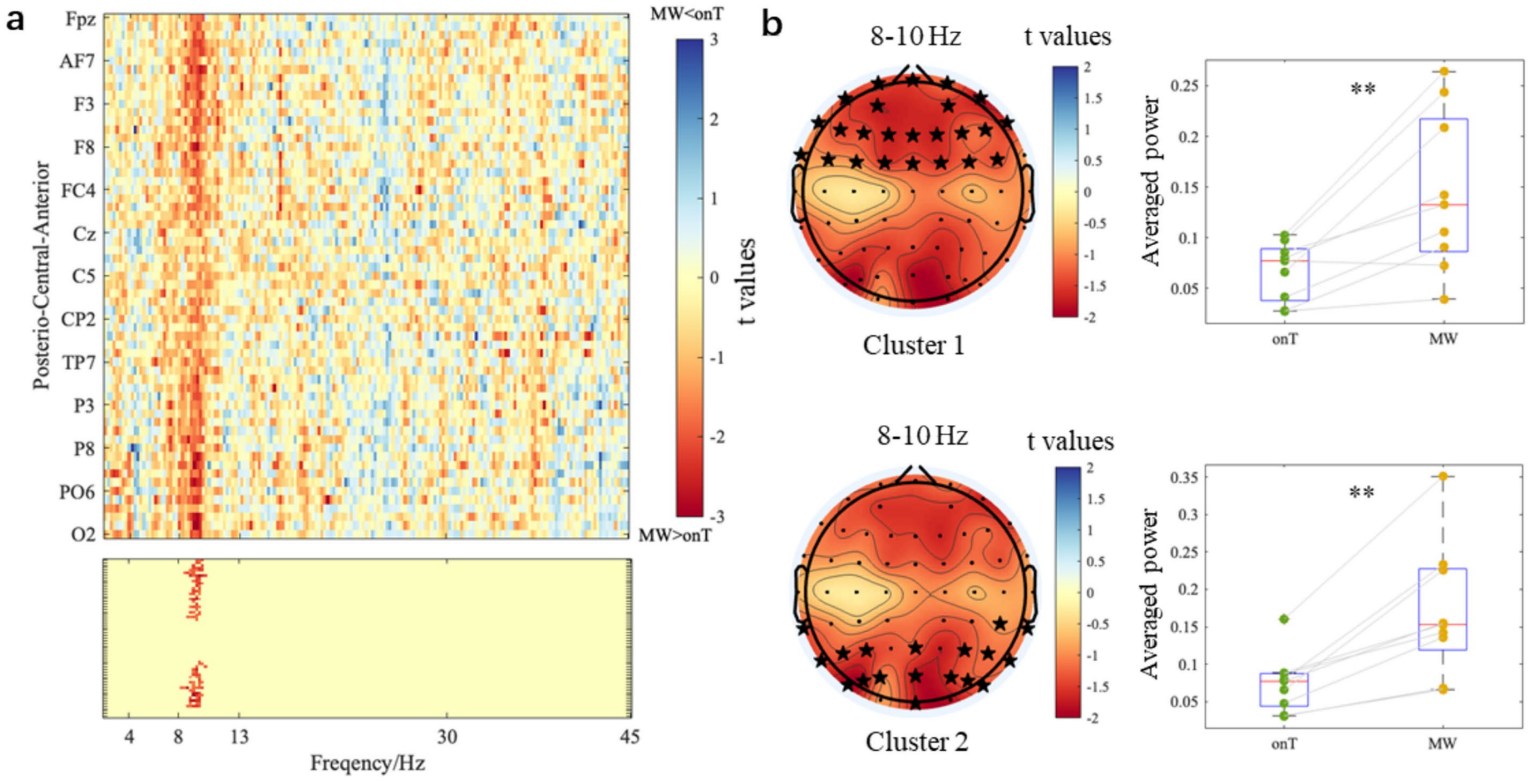
Power differences between onT and MW conditions. **(a)** The top panel presents the t-value maps from paired-sample *t*-tests (onT minus MW) for each frequency point and each electrode. The bottom panel represents two significant clusters (*p*-cluster <0.025) at 8-10 Hz. **(b)** The left panels present scalp topographies of 8-10 Hz *t*-values. Electrodes that survived from the permutation test were marked with black stars. The right panels show cluster-averaged power within 8–10 Hz under onT and MW conditions. **p < 0.001.

For the trials classified as onT and MW, power spectra were estimated at each electrode for every frequency point between 2 and 45 Hz in a step of 0.1 Hz. Paired-sample *t*-tests were conducted to assess the power differences between conditions, with t-values (onT minus MW) visualized as spatial-frequency topographies (Figure 4a). Cluster-based permutation tests were then applied, and *t*-values that survived the significance threshold (*p*-cluster <0.025) are shown in the lower panel of Figure 4a. The analysis revealed two significant clusters in the low alpha band (8–10 Hz). Cluster 1 was primarily distributed over the frontal region, encompassing electrodes FPz, Fz, FCz, FP1, AF7, AF3, F1, F3, F5, F7, FC1, FC3, FC5, FT7, FP2, AF8, AF4, F2, F4, F6, F8, FC2, FC4, and FC6. Cluster 2 was mainly distributed over the posterior region, involving electrodes POz, Oz, Pz, TP7, P7, P5, P3, PO7, PO5, PO3, TP8, CP6, P4, P6, P8, PO4, PO6, and PO8. Additionally, cluster-averaged power within the 8–10 Hz band was compared between the two conditions. As shown in Figure 4b, the cluster-averaged power during the MW condition was significantly higher than during the onT condition in both the frontal region (MW = 0.142 ± 0.079, onT = 0.068 ± 0.029; *p* < 0.001) and the posterior region (MW = 0.170 ± 0.089, onT = 0.074 ± 0.039; *p* < 0.001).

**Figure 5:**
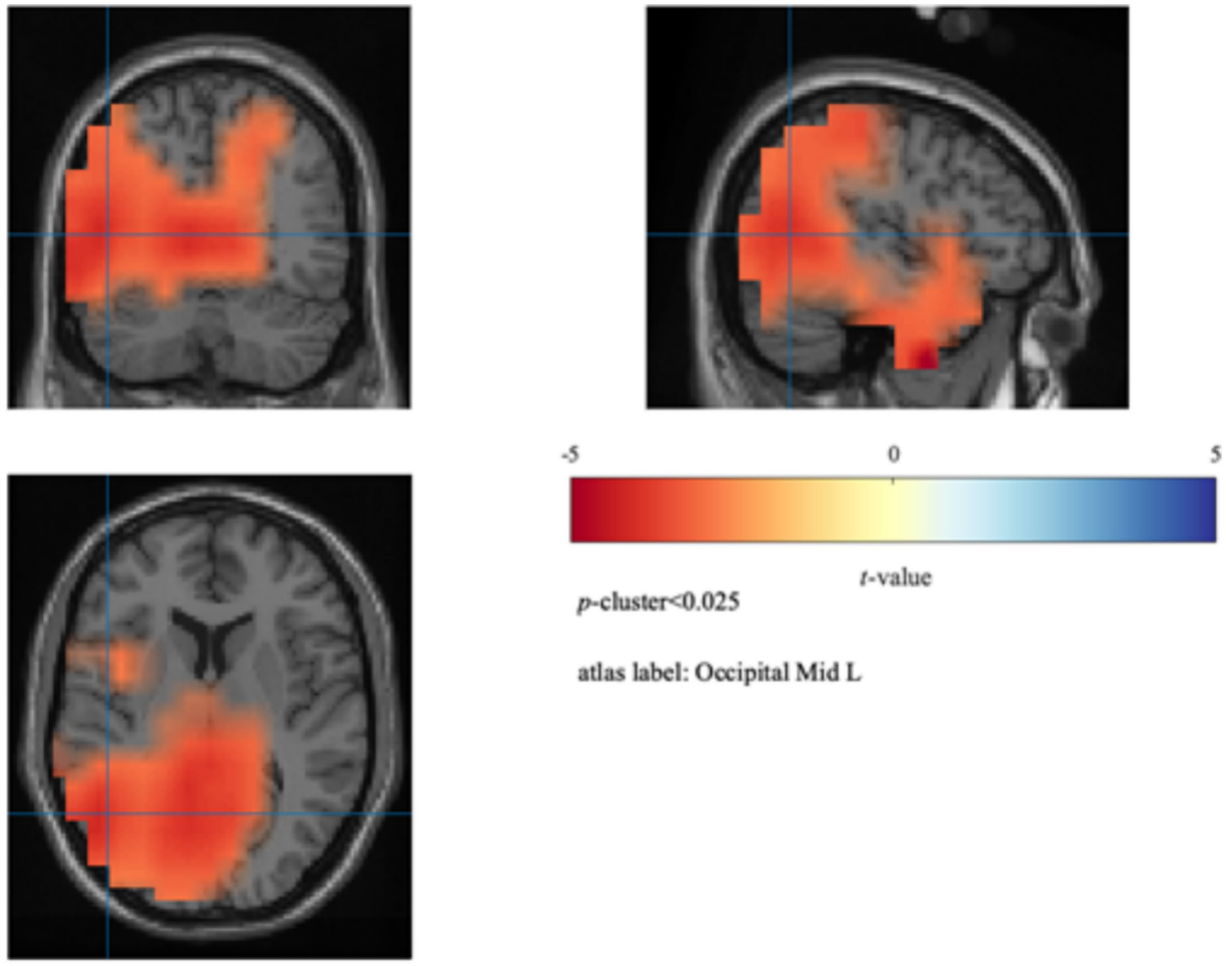
Source localization results. Beamformer analysis showing increased 8–10 Hz power during MW relative to onT in the left middle occipital gyrus (MNI [−30–70 12]).

To further localize the cortical sources associated with attentional fluctuations during the force control task, we conducted source localization analysis using a beamformer algorithm implemented in FieldTrip (Oostenveld et al., 2011). For each participant, source activity estimates were obtained for both the onT and MW conditions within the 8–10 Hz band. Paired-sample *t*-tests were used to assess differences in neural activity between the two conditions, followed by cluster-based permutation testing (*p*-cluster <0.025). As shown in Figure 5, the average power in the left middle occipital gyrus (MNI: [−30–70 12]) was significantly higher during the MW condition (3.51 ± 0.39 dB) than during the onT condition [3.36 ± 0.40 dB; *t*(8) = 4.99, *p* = 0.0084].

### 3.3 Condition-specific differences in alpha-theta ratios

**Figure 6:**
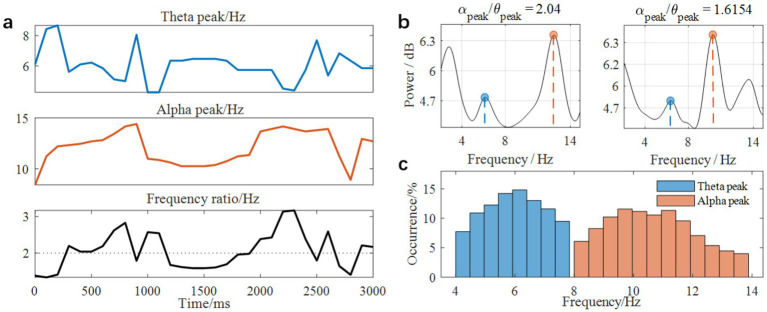
Peak frequency detection of alpha and theta bands and ratio calculation. **(a)** Temporal variability of peak frequencies and ratios for an exemplary participant and electrode. **(b)** Spectral power in two exemplary trials: alpha and theta peak frequencies formed a harmonic (ratio = 2.04) versus a non-harmonic (ratio = 1.62) relationship. **(c)** Distribution of peak frequencies across trials.

Synchronization between neural oscillations at different frequencies has been proposed as a core mechanism for the coordination and integration of neural systems. Mathematically, when two oscillators with different frequencies form a harmonic relationship (e.g., *f*_1_/*f*_2_ = 2), as opposed to a nonharmonic relationship (e.g., *f*_1_/*f*_2_ = 1.6), the harmonic arrangement allows for more frequent excitatory phase meetings, thereby facilitating cross-frequency synchronization. In line with this principle, recent theoretical frameworks suggest that shifts in oscillatory peak frequencies constitute a principal mechanism for implementing cross-frequency coupling and decoupling in the brain (Rodriguez-Larios and Alaerts, 2019, 2021; Rodriguez-Larios et al., 2020). Following this framework, we quantified cross-frequency coupling by analyzing peak frequency ratios between different bands and compared them between onT and MW conditions during the force control task. Given the established role of alpha-theta coupling in tasks involving attention and executive control, we specifically examined peak frequency ratios between the alpha and theta bands. Figure 6a illustrates trial-wise variability in alpha and theta peak frequencies, as well as their corresponding numerical ratios over a 3-s period for a representative participant and electrode. Figure 6b compares the spectral power in two representative trials: one showing a harmonic alpha-theta ratio (2.04) and the other a non-harmonic ratio (1.62). Figure 6c shows the distribution of alpha and theta peak frequencies across all trials for the same participant and electrode.

**Figure 7:**
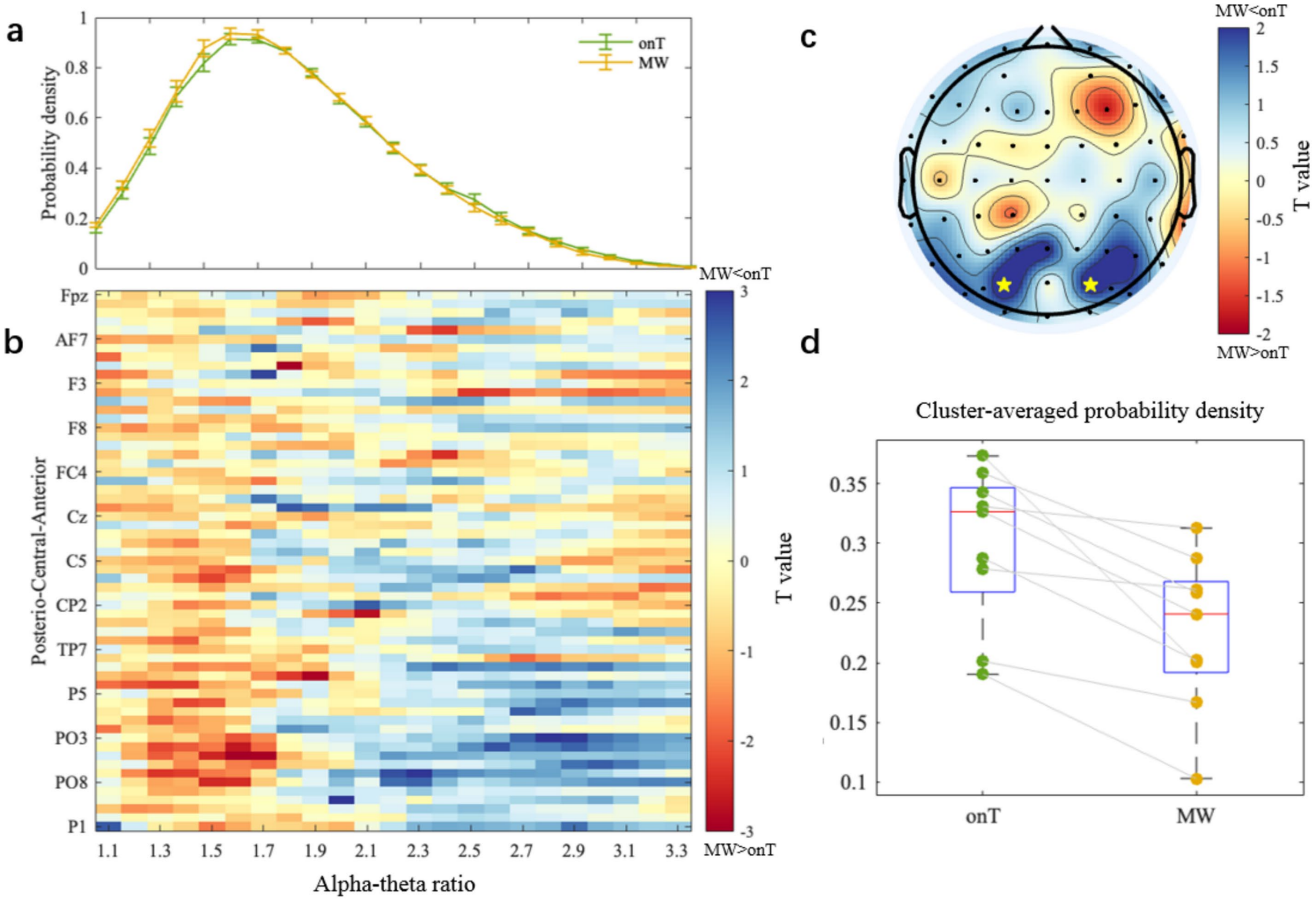
Condition-specific differences in alpha-theta ratios. **(a)** Probability density of each alpha-theta ratio (from 1.1 to 3.3 with a step of 0.1) averaged across electrodes and subjects. Error bars represent standard deviation across subjects. **(b)** *t*-value map of condition differences (onT minus MW). Positive *t*-values shown in cold colors indicate higher probability density under onT condition compared to MW condition. **(c)** Topographical heat map of t-values averaged over 2.6–3.0 ratios. Two electrodes (PO3 and PO4) showing significance during the cluster-based permutation statistics are marked by yellow stars. **(d)** Cluster-averaged probability density under onT and MW conditions.

Differences between onT and MW conditions were further assessed using paired-sample *t*-tests for each electrode and cross-frequency ratio (ranging from 1.1 to 3.3 in a step of 0.1). Cluster-based permutation testing was applied to identify significant clusters based on adjacency in electrode space and cross-frequency ratio, while controlling for multiple comparisons. As described in Section 2.4.2.2, the distribution of alpha-theta ratios was represented by its probability density, where higher density at a given ratio reflects a greater likelihood of oscillatory alignment around that harmonic relationship. Figure 7a shows the probability density of each ratio averaged across all electrodes for the onT and MW conditions. Figure 7b visualizes the condition differences in probability density by plotting t-values for each ratio and electrode. Positive *t*-values (shown in cold colors) indicate higher probability density during the onT condition compared to the MW condition. A negative cluster (i.e., MW > onT) was observed at posterior electrodes within the lower ratio range (1.2–1.7), although it did not reach significance in the permutation test. However, a significant positive cluster was identified within the 2.6–3.0 ratio range, indicating a significantly higher probability density during the onT condition compared to the MW condition (*p*-cluster < 0.05). Figure 7c presents the topographical heat map of t-values averaged over the 2.6–3.0 ratio range, with PO3 and PO4 marked as significant electrodes from the permutation test. A repeated one-way ANOVA on the identified cluster confirmed that the cluster-averaged probability density within the 2.6–3.0 ratio range was significantly higher during the onT condition (0.299 ± 0.066) than during the MW condition [0.226 ± 0.065; *F*_(1,92)_ = 8.21, *p* = 0.0005; Figure 7d].

### 3.4 Condition-specific differences in functional connectivity

**Figure 8:**
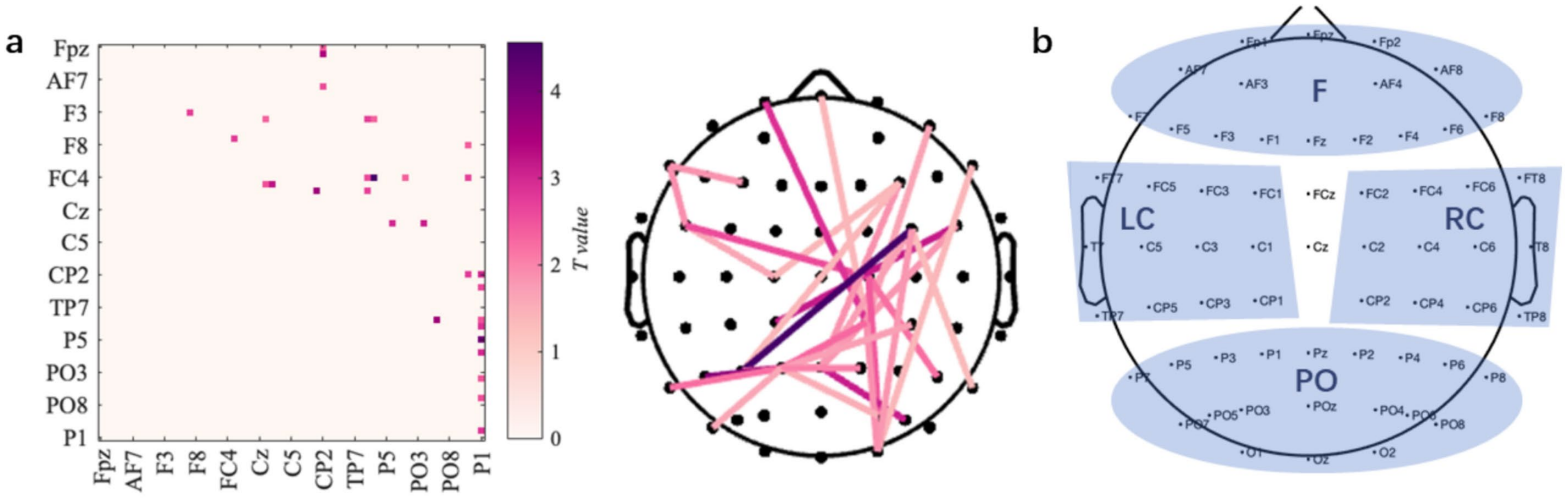
Functional connectivity differences between onT and MW conditions. **(a)** The left panel illustrates the differences in PLV between the onT and MW conditions by plotting paired-sample *t*-test values. The right panel highlights the connections showing significant increases during MW compared to onT. **(b)** Four electrode categories used for the community connection analysis: F (frontal lobe), PO (parieto-occipital lobe), LC (left central motor area), and RC (right central motor area).

Given the significant condition-related differences observed in the 8–10 Hz band (Section 3.2), we analyzed PLVs between all electrode pairs to investigate functional connectivity differences within this frequency range. Paired-sample *t*-tests with permutation-based correction revealed no significant differences for the onT > MW comparison, but significantly higher connectivity during MW compared to onT at specific connections. The statistically significant PLV values and their corresponding electrode pairs are shown in Figure 8a.

To investigate condition-related connectivity differences from a network-level perspective, electrodes were grouped into four brain regions (Figure 8b): frontal lobe (F), parieto-occipital lobe (PO), left central motor area (LC), and right central motor area (RC). Community connectivity (CC) was computed by averaging PLVs within or between these defined regions. A repeated-measures one-way ANOVA revealed significantly higher intra-region CC within the LC during the MW condition compared to the onT condition [*F*_(1,100)_ = 19.76, *p* < 0.001]. Inter-region CC between PO-RC [*F*_(1,100)_ = 9.36, *p* = 0.003], F-LC [*F*_(1,100)_ = 6.84, *p* = 0.010], and LC-RC [*F*_(1,100)_ = 6.85, *p* = 0.010] was also significantly higher during the MW condition than during the onT condition. Descriptive statistics and corresponding significance values are summarized in Table 1.

**Table 1:** Descriptive and statistics results of community connection analysis.

Brain regions	CC-onT (±SD)	CC-MW (±SD)	*F*-values	*p*-values
F	0.681 ± 0.035	0.675 ± 0.083	0.015	0.904
LC	0.617 ± 0.053	0.668 ± 0.077	19.761	2.28e-5***
RC	0.606 ± 0.055	0.615 ± 0.107	0.120	0.729
PO	0.656 ± 0.052	0.643 ± 0.063	2.817	0.096
F-LC	0.537 ± 0.035	0.566 ± 0.087	6.843	0.010*
F-RC	0.542 ± 0.035	0.559 ± 0.087	1.928	0.168
F-PO	0.605 ± 0.037	0.610 ± 0.063	0.138	0.711
LC-RC	0.541 ± 0.051	0.570 ± 0.086	6.848	0.010*
LC-PO	0.520 ± 0.031	0.536 ± 0.075	0.858	0.357
RC-PO	0.524 ± 0.029	0.565 ± 0.073	9.362	0.003**

*p < 0.05, **p < 0.01, ***p < 0.001.

### 3.5 Condition-specific differences in neural-behavioral synchronization

**Figure 9:**
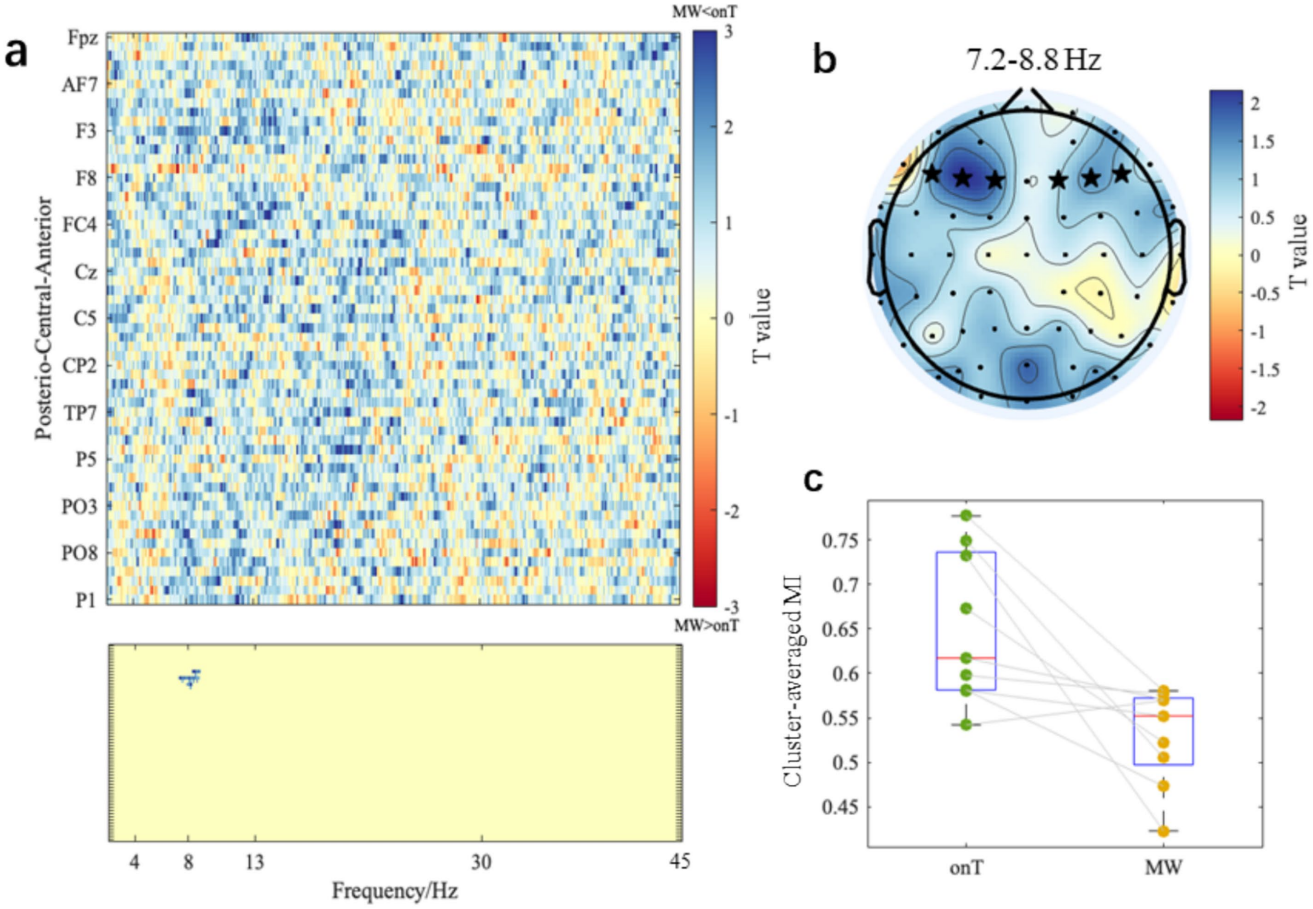
Neural–behavioral synchronization differences between onT and MW conditions. **(a)** The top panel displays the paired-sample *t*-test values per electrode and frequency point (2–45 Hz in 0.1 Hz step). The bottom panel represents the cluster that survived from the cluster-based permutation test (*p*-value of cluster <0.05). **(b)** Scalp topographical map of *t*-values averaged within 7.2–8.8 Hz, with electrodes that survived the cluster-based permutation test marked by black stars. **(c)** Cluster-averaged MI within 7.2–8.8 Hz under onT and MW conditions.

Mutual information (MI) between the within-trial force error (*BE*_NL_) and EEG power amplitude was analyzed to assess neural-behavioral synchronization. Figure 9a presents paired-sample *t*-test values for each electrode and frequency point (2–45 Hz in 0.1 Hz steps), where positive *t*-values (shown in cold colors) indicate higher MI during the onT condition compared to the MW condition. Cluster-based permutation testing identified a significant negative cluster within the 7.2–8.8 Hz range (*p*-cluster < 0.05), indicating significantly stronger neural-behavioral synchronization during the onT condition compared to the MW condition. This negative cluster was located in the anterior region, including electrodes F1, F3, F5, F2, F4, and F6. Figure 9b shows the topographical *t*-value map averaged across these six electrodes within the 7.2–8.8 Hz range. A repeated-measures one-way ANOVA on the identified cluster further confirmed significantly higher MI during the onT condition (0.650 ± 0.085) compared to the MW condition [0.530 ± 0.054; *F*_(1,92)_ = 10.69, *p* = 0.0015; Figure 9c].

### 3.6 Attentional states classification results

An SVM-RBF model was trained to classify binary attentional states and evaluated using two cross-validation strategies (i.e., LOSO and 5-fold cross-validation). Each feature that passed the aforementioned statistical tests was evaluated individually in separate classification models. Table 2 summarizes the classification performance metrics for each individual feature under both validation strategies. The chance-level accuracy is 50% because the dataset was balanced using the quartile-based labeling approach. Here, MW was defined as the positive class. The single-feature models showed generally comparable classification performance across both validation strategies. Among the four features, spectral power yielded the highest accuracy, precision, F1-score, and AUC. Considering comparability with existing literature, this study primarily focuses on the accuracy and AUC metrics. For the cross-participant classification (i.e., LOSO strategy), the power feature achieved a mean accuracy of 66.99 ± 12.34% and a mean AUC of 76.37 ± 19.26%. Similarly, in the within-participant classification (i.e., 5-fold strategy), the power feature yielded a mean accuracy of 68.28 ± 7.14% and a mean AUC of 71.86 ± 7.05%. For the MI-only model, the recall for the MW class was high, whereas overall accuracy, precision, and AUC remained low. This indicates a bias toward predicting MW trials, leading to numerous false positives for onT trials, as illustrated by the confusion matrices shown in Supplementary Figure 3.

**Table 2:** Classification performance using individual features under LOSO and 5-fold cross-validation (Mean ± SD).

Metrics	Spectral power	Ratio	PLV	MI
LOSO cross validation (%)				
Accuracy	**66.99 ± 12.34**	54.07 ± 12.89	54.26 ± 14.32	50.42 ± 7.72
Recall	80.00 ± 27.44	33.52 ± 22.46	41.39 ± 30.47	94.54 ± 8.41
Precision	64.48 ± 11.18	57.41 ± 30.17	53.70 ± 28.29	50.25 ± 5.43
F1-score	68.75 ± 17.67	40.04 ± 20.83	43.27 ± 25.09	65.59 ± 5.43
AUC	**76.37 ± 19.26**	61.65 ± 14.90	51.83 ± 22.61	45.47 ± 14.93
5-fold cross validation (%)				
Accuracy	**68.28 ± 7.14**	54.91 ± 9.99	51.32 ± 6.37	50.09 ± 6.03
Recall	81.35 ± 10.31	42.22 ± 25.03	40.97 ± 33.58	90.26 ± 9.74
Precision	65.27 ± 7.52	49.12 ± 28.21	50.02 ± 13.37	50.09 ± 3.32
F1-score	71.92 ± 5.64	44.88 ± 25.38	40.39 ± 21.46	64.32 ± 4.64
AUC	**71.86 ± 7.05**	61.43 ± 9.49	49.66 ± 11.17	47.78 ± 10.46

Bold values indicate the best performance in terms of Accuracy and AUC.

To evaluate whether synchronization features (i.e., ratio, PLV, and MI) could enhance the classification performance, we combined the spectral power feature with these synchronization features as inputs to the SVM-RBF models. Table 3 presents the classification results from all possible combinations of spectral power and synchronization-related features. Overall, combining spectral power with any of the synchronization features improved classification accuracy and AUC. Under LOSO cross-validation, the combination of spectral power, ratio, and PLV achieved the highest accuracy (71.57 ± 10.79%) and AUC (79.87 ± 15.59%). In 5-fold cross-validation, the combination of all four features yielded the highest accuracy (75.53 ± 8.40%), while the combination of spectral power, ratio, and PLV achieved the highest AUC (76.58 ± 7.07%).

**Table 3:** Classification performance using feature combinations under LOSO and 5-fold cross-validation (mean ± SD).

Metrics	Accuracy	Recall	Precision	F1-score	AUC
LOSO cross validation (%)					
Power + MI	69.21 ± 14.55	66.39 ± 31.00	68.58 ± 30.96	64.55 ± 25.87	77.51 ± 11.34
Power + PLV	71.02 ± 15.24	67.78 ± 32.32	71.16 ± 32.37	66.21 ± 26.80	77.88 ± 15.79
Power + Ratio	70.46 ± 12.59	62.96 ± 33.85	74.45 ± 33.41	63.35 ± 26.01	79.29 ± 14.84
Power + Ratio +PLV	**71.57 ± 10.79**	67.04 ± 28.89	83.34 ± 18.39	67.70 ± 17.13	**79.87 ± 15.59**
Power + Ratio + MI	68.10 ± 13.36	67.04 ± 28.89	79.87 ± 221.71	65.67 ± 16.94	76.63 ± 16.32
Power + PLV + MI	67.82 ± 16.42	68.24 ± 30.39	66.85 ± 31.36	64.64 ± 25.85	77.44 ± 13.03
Power + Ratio + PLV + MI	70.37 ± 13.79	77.41 ± 24.82	76.20 ± 18.67	71.42 ± 12.79	78.34 ± 14.53
5-fold cross validation (%)					
Power + MI	72.21 ± 12.74	71.44 ± 17.12	72.50 ± 12.36	71.60 ± 13.26	75.20 ± 7.05
Power + PLV	71.21 ± 12.16	71.44 ± 17.12	70.56 ± 9.94	70.79 ± 12.90	74.56 ± 8.24
Power + Ratio	73.43 ± 10.34	70.97 ± 11.06	75.29 ± 11.41	72.79 ± 9.81	76.42 ± 7.16
Power + Ratio + PLV	71.43 ± 8.31	75.20 ± 11.27	69.97 ± 7.46	72.31 ± 8.38	**76.58 ± 7.07**
Power + Ratio + MI	71.09 ± 7.19	73.83 ± 13.18	70.60 ± 6.76	71.55 ± 7.64	75.07 ± 6.26
Power + PLV + MI	71.97 ± 11.17	76.51 ± 14.24	69.88 ± 8.77	72.94 ± 11.03	74.39 ± 7.59
Power + Ratio + PLV + MI	**75.53 ± 8.40**	80.50 ± 5.43	70.87 ± 7.32	75.23 ± 5.62	76.12 ± 5.81

Bold values indicate the best performance in terms of Accuracy and AUC.

## 4 Discussion

This study aimed to advance the understanding of the neurophysiological signatures and detection of mind wandering (MW) by investigating EEG synchronization features within a novel visuo-haptic force control paradigm. Although MW has been extensively studied in visual and auditory domains, its neural correlates during sensorimotor engagement—particularly involving force modulation—remain largely unexplored. Moreover, despite the theoretical importance of neural synchronization in attention regulation, few studies have systematically assessed the efficacy of EEG-based functional connectivity (FC), cross-frequency coupling (CFC), and neural-behavioral synchronization (NBS) in detecting MW, especially in haptic contexts. Our investigation yielded key insights into the neural signatures of MW during force control and demonstrated the feasibility of using synchronization features for attentional state classification.

### 4.1 Neural signatures of mind wandering

The proposed continuous force control task effectively induced MW episodes, as evidenced by a significant upward trend in off-task ratings over trials and a marked degradation in behavioral performance (i.e., increased BE_NL-trial_ errors) during the MW condition compared to the onT condition. In terms of neural activity, we observed increased alpha power (8–10 Hz) over frontal and parieto-occipital regions during MW, with source localization revealing the increased activity in the left middle occipital gyrus. This widespread alpha power increase during MW aligns with the “perceptual decoupling” hypothesis (Schooler et al., 2011; Smallwood, 2013) and numerous EEG studies using visual vigilance tasks such as the SART (Compton et al., 2019; Jin et al., 2019). Notably, our findings are consistent with recent observations by Luna et al. (2023), who reported increased alpha power in left occipital regions prior to missed targets compared to correct detections in a visual vigilance task, suggesting that alpha increases may reflect attentional disengagement across both visual and visuo-motor tasks. Regarding the haptic domain, while Peng et al. (2021) reported increased frontal-central alpha associated with off-task states in a discrete force task, we observed a more distributed pattern involving both frontal and posterior areas. The localized increase in the left middle occipital gyrus indicates that even in a force-focused task, MW involves disengagement of visual processing regions, likely reflecting the visuo-haptic integration demands of our paradigm, since force adjustments in our task were guided by visual feedback. These findings highlight the modality-independent nature of alpha increases as a potential neural marker of attentional disengagement.

Beyond single-frequency analyses, we evaluated cross-frequency coupling and found significantly reduced probability density of high alpha-theta ratios (2.6–3.0) under the MW state, particularly over parietal-occipital electrodes PO3 and PO4. While prior work by Rodriguez-Larios and Alaerts (2019, 2021) and Rodriguez-Larios et al. (2020) linked increased alpha-theta phase synchrony to MW during meditation, our focus on harmonic frequency ratios reveals a different aspect of cross-frequency organization. Harmonic ratios near 3.0 may support on-task attention by enabling stable cross-frequency phase coupling. This precise phase alignment likely optimizes communication between neural assemblies supporting top-down control (theta) and those involved in sensory inhibition (alpha), thereby facilitating efficient information integration. The reduction of these stable harmonic ratios during MW suggests a breakdown in this coordinated cross-frequency mechanism. This view aligns with theories proposing that optimal cognitive control relies on harmonic cross-frequency arrangements enabling effective communication between neural assemblies (Fries, 2005; Palva and Palva, 2017). The parietal-occipital localization (PO3/PO4) further implicates visuospatial processing networks in maintaining this rhythmic coordination during force tracking. Nevertheless, whether the near 3.0 ratios observed here also support focused attention in other motor tasks or in other modalities (such as pure visual or auditory tasks) remains to be determined by future studies. Direct cross-task comparisons and source-level CFC analyses would be required to assess the generalizability.

Additionally, MW was associated with enhanced functional connectivity across sensorimotor networks. Within the 8–10 Hz band, MW states exhibited significantly stronger phase locking within the left central motor area and between the PO-RC, F-LC, and LC-RC communities. One plausible interpretation is the compensatory recruitment of task-relevant sensorimotor assemblies when top-down control wanes; such localized synchronization could transiently support baseline performance despite attentional lapses. This finding extends previous fMRI research highlighting the dominance of the DMN during mind wandering by suggesting a context-dependent compensatory mechanism: in the force control task requiring continuous sensorimotor engagement, attenuated top-down attention may trigger localized synchronization within task-relevant networks to sustain baseline performance (Christoff et al., 2009). This aligns with recent findings demonstrating a dynamically interdependent relationship between external (sensory and motor processing) and internal cognition (mind wandering) (Long et al., 2025). In accordance with the recently proposed “Baseline model of internal and external cognition” (Northoff et al., 2022), the observed hyperconnectivity likely reflects inefficient neural resource reallocation—where heightened “noisy” processing in sensorimotor circuits fails to fully compensate for attentional lapses—as evidenced by concurrent increases in behavioral errors. Our results underscore that MW dynamically redistributes neural resources with sensorimotor synchronization representing a signature of embodied attentional fluctuations. Nevertheless, alternative explanations for increased sensor-level connectivity—such as contamination by muscle activity, volume conduction, field spread, or other recording artifacts—cannot be ruled out (Haufe et al., 2012). Importantly, our connectivity estimates were derived at the sensor level without full source-level leakage correction; therefore, these results should be interpreted with caution as preliminary evidence. Future studies should complement sensor-level FC with source reconstruction, leakage-robust metrics, and muscle activity monitoring to better distinguish neural coupling from confounds.

In addition to neural synchronization metrics, we also examined the coupling between neural activity and behavioral performance by assessing the mutual information between the force error (i.e., BE_NL_) and EEG power. During MW, the MI was significantly reduced within the 7.2–8.8 Hz band over several frontal electrodes, suggesting a breakdown in the real-time coupling between brain dynamics and motor output during attentional lapses. This observation is conceptually novel. Although previous studies have linked behavioral variability (e.g., RT variability) to MW (Esterman et al., 2013; Peng et al., 2021), none have quantified the dynamic synchronization between continuous neural signals and high-frequency motor performance. The 7.2–8.8 Hz band overlaps with the low-alpha/mu rhythm, known to reflect motor cortical excitability and somatosensory processing. Reduced NBS likely reflects a weakened predictive relationship between fluctuations in this rhythm and moment-to-moment force control accuracy weakens during MW. This decoupling might provide an objective neurobehavioral signature of attentional disengagement specific to active motor tasks and represents an advance beyond static behavioral error measures.

### 4.2 Classification performance

The SVM classification achieved optimal performance when traditional power features were combined with synchronization metrics. Within-participant models (5-fold CV) using all features reached 75.53% accuracy (AUC = 76.12%), while cross-participant models (LOSO CV) using power + alpha-theta ratio + FC features achieved 71.57% accuracy (AUC = 79.87%). These results are comparable to or slightly better than performance reported in similar binary classification studies (Jin et al., 2019, 2020; Dong et al., 2021; Chen et al., 2022). Several factors likely contributed to the classification performance. First, the combination of commonly used features (i.e., power) and novel synchronization features (cross-frequency ratio, FC, NBS) offered complementary information. Notably, adding synchronization features consistently boosted performance over power features alone (e.g., LOSO AUC increased from 76.37 to 79.87% with power + ratio + FC), highlighting the value of capturing distributed network dynamics and brain-behavior interactions. Second, the proposed continuous force task provided a rich stream of behavioral data (1,000 Hz) tightly synchronized with EEG data. This enabled the calculation of NBS, a feature unavailable in discrete response tasks and proved to be a useful feature for classification. Third, the continuous, dynamic nature of the force control task likely elicited more pronounced and ecologically valid MW states compared to simpler vigilance tasks, leading to clearer neural dissociations.

However, direct comparisons are challenging due to differences in tasks, probing methods, and classification approaches (e.g., LOPOCV vs. LOSO vs. within-participant). Our cross-participant accuracy (71.57%) highlights the challenge of generalizing models across individuals, a common limitation in the field. Future work should explore more advanced normalization or domain adaptation techniques.

### 4.3 Limitations and future work

While this study offers novel insights, the findings should be considered with several limitations. First, the final sample size of nine participants (after artifact rejection) is relatively small and all participants were male. Future studies should recruit larger, more diverse cohorts, including females and individuals from broader age ranges, to enhance generalizability and to explore potential sex differences in neural correlates of MW during motor tasks. Second, while synchronization features improved classification, their neurobiological interpretation remains complex. For instance, increased FC within motor areas during MW could reflect different processes (e.g., maladaptive noise, inefficient compensation, or muscle artifacts). Given the sensor-level nature of our connectivity analyses, we emphasize the need for source-space reconstructions and leakage-robust measures in future work to distinguish true inter-region interactions from sensor-level mixing. Future research should also integrate computational modeling or causal interventions (e.g., transcranial magnetic stimulation) to elucidate the functional significance of these connectivity patterns. Third, EEG epochs were extracted from the 3 s preceding thought-probes, which occurred at 40–50 s intervals. This quasi-periodicity could have induced anticipatory effects or strategic attention re-engagement just before probes, potentially affecting the MW vs. onT contrast. However, the overall monotonic increase in MW reports across trials, coupled with the corresponding behavioral degradation, suggests that anticipatory effects alone are unlikely to fully account for our main findings. Nevertheless, future studies are necessary to employ randomized probe timing to eliminate this potential confound.

Finally, our findings pertain to a specific visuo-haptic force control task. The generalizability of the identified synchronization signatures to other haptic tasks or modalities remains to be investigated. Future studies should systematically compare MW signatures across different task types, such as pure haptic vs. visuo-haptic or force vs. texture discrimination. Future studies should also explore more sophisticated synchronization measures, such as directed connectivity (Tafreshi et al., 2019), graph theory metrics (Moon et al., 2020), and advanced machine learning algorithms that may better capture complex spatiotemporal dynamics in EEG data.

## 5 Conclusion

This study demonstrates that EEG synchronization features—including functional connectivity within sensorimotor networks, alterations in alpha-theta cross-frequency coupling, and neural–behavioral synchronization—serve as sensitive and complementary markers of mind wandering during a continuous visuo-haptic force control task. Our findings extend the understanding of attentional fluctuations beyond visual/auditory paradigms, revealing task-specific neural dynamics characterized by increased motor network synchrony and disrupted neural-behavioral alignment during attentional lapses. Using these synchronization features, machine learning classifiers achieved 75.53% within-participant and 71.57% cross-participant accuracy when combined with spectral power features, confirming their feasibility and complementary value for decoding covert attentional states. This work extends the understanding of neural representations underlying attentional fluctuations during the continuous force control process. While our paradigm was constrained to a laboratory setting, the identified synchronization markers may provide preliminary insights to guide the future development of haptic-based attention training systems. Such systems could serve as a complementary approach to existing visual and auditory modalities in contexts such as sports training, surgical skill learning, or neurorehabilitation, pending further validation in more ecologically valid settings.

## Data Availability

The raw data supporting the conclusions of this article will be made available by the authors, without undue reservation.
