# Supplementary material for: EEG synchronization signatures for decoding attentional states during continuous force control
Source: Front Neurosci. 2025 Oct 8;19:1654827. doi: 10.3389/fnins.2025.1654827 (PMC12540398; doi:10.3389/fnins.2025.1654827)
Supplement: Supplementary file 1 [file Data_Sheet_1.docx]

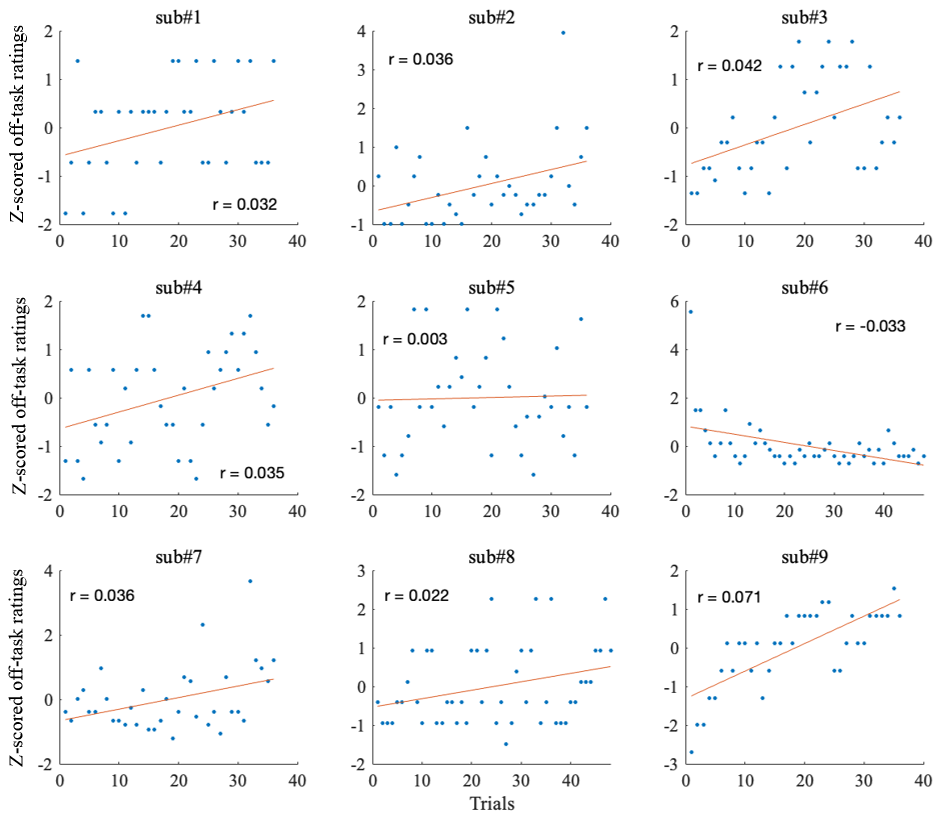


**Supplementary Figure 1.** Z-scored off-task ratings of all trials (blue dots) and the regression-fitted curve (red line) for all subjects.


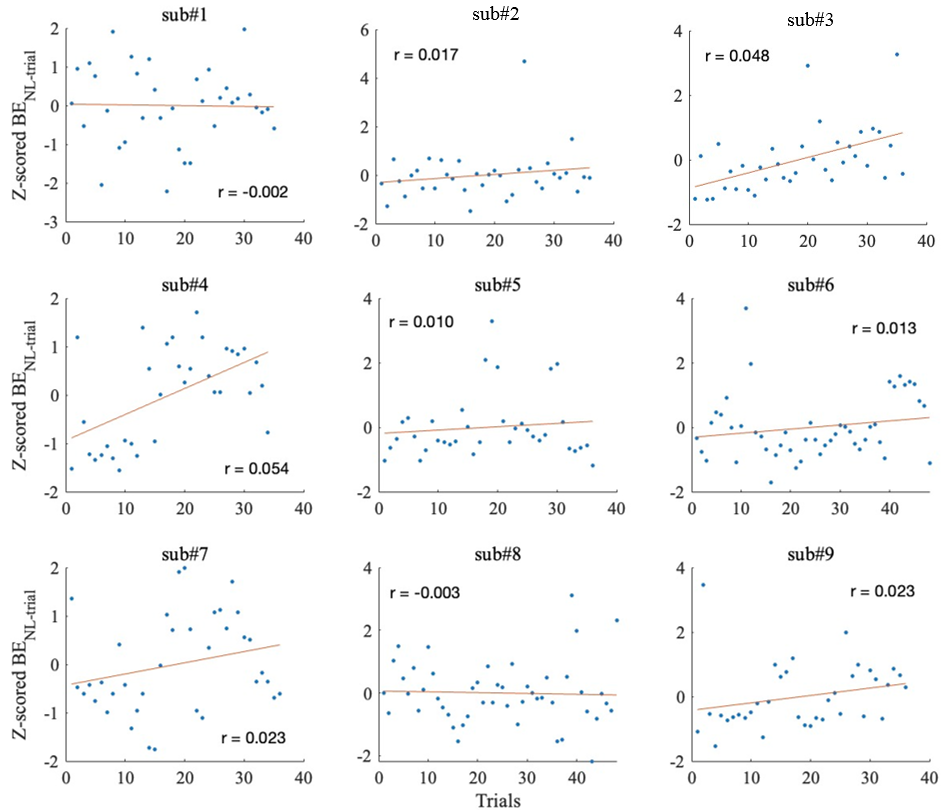


**Supplementary Figure 2.** Z-scored BE_NL-trial_ of all trials (blue dots) and the regression-fitted curve (red line) for all subjects.


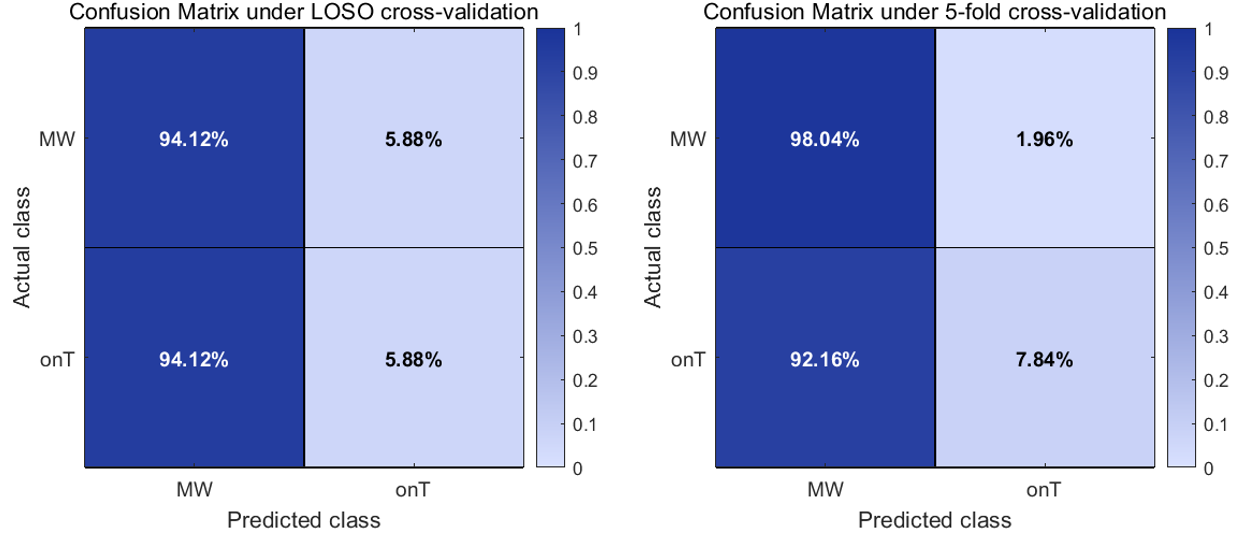


**Supplementary Figure 3.** Confusion matrices of MI-only model under leave-one-subject-out (LOSO) and 5-fold cross-validation.
